# The updated relationship between the cleft‑lip and palate transmembrane protein‑1‑like rs401681 and lung cancer risk: A systematic review and meta‑analysis

**DOI:** 10.3892/mco.2024.2768

**Published:** 2024-07-31

**Authors:** Zemin Fang, Gaofeng Zhao, Yuebin Wang, Fengke Li, Zhidan Ding

**Affiliations:** Department of Lung Transplant, The First Affiliated Hospital of Zhengzhou University, Zhengzhou, Henan 450000, P.R. China

**Keywords:** cleft-lip and palate transmembrane protein-1-like, rs401681, polymorphism, lung cancer risk, meta-analysis

## Abstract

Currently, the role of cleft-lip and palate transmembrane protein-1-like (CLPTM1L) rs401681 in various tumor types, particularly lung cancer, has garnered significant attention. However, the findings across studies have shown discrepancies. The aim of the present meta-analysis was to provide a more nuanced understanding of the involvement of CLPTM1L rs401681 in lung cancer development. Several electronic databases were systematically searched, including PubMed, Cochrane Library, Embase, Medline, Wanfang, Google Scholar and Chinese National Knowledge Infrastructure. Odds ratios (ORs) and 95% confidence intervals (CIs) were synthesized using random-effects models. Heterogeneity of included studies was assessed using the I^2^ statistic and Q test. Sensitivity analysis was conducted to evaluate the stability of overall estimates. Moreover, Egger's test was utilized to detect potential publication bias. The collective ORs indicated a significant association between the CLPTM1L rs401681 polymorphism and susceptibility to lung cancer across various genetic comparisons. These encompass allele T vs. allele C (OR=0.93, 95% CI=0.88-0.99, P<0.001), TT + CT vs. CC (OR=0.91, 95% CI=0.87-0.96, P<0.001), TT vs. CC + CT (OR=0.88, 95% CI=0.80-0.96, P<0.001), TT vs. CC (OR=0.84, 95% CI=0.75-0.94, P<0.001) and CT vs. CC (OR=0.84, 95% CI=0.75-0.94, P<0.001). Examination through statistical Q test and I^2^ statistic revealed pronounced heterogeneity across four genetic comparisons (allele T vs. allele C, TT + CT vs. CC, TT vs. CC and CT vs. CC). Ethnical distinctions emerged as the primary, if not exclusive, sources of the significant heterogeneity. Upon stratification by ethnicity, a notable reduction in heterogeneity was discernible within the Caucasian demographic. However, heterogeneity persisted within the Asian population. Furthermore, lung cancer risks were statistically significantly decreased for individuals possessing allele T through all genetic comparisons within Caucasians; whereas among Asians, significant reduction was observed solely in the TT vs. CC comparison. The present meta-analysis uncovers a significant association between the CLPTM1L rs401681 polymorphism and altered susceptibility to lung cancer.

## Introduction

Lung cancer currently ranks among the foremost causes of cancer death, with its incidence and fatality rates escalating annually (1). In 2012, ~1.8 million new cases of lung cancer were reported globally, resulting in 1.6 million fatalities (1). Epidemiological data suggest an estimated 230,000 new cases of lung cancer in the USA in 2022. Furthermore, the mortality rate attributed to lung cancer is anticipated to surpass the combined rates of breast, prostate and colon cancer (2). These conditions impose substantial economic and psychological burdens on society and families. While smoking is a well-established risk factor for lung cancer, accounting for 80-90% of the cases, it is noteworthy that only 15% of smokers ultimately develop lung cancer. This suggests that hereditary predisposition might also significantly contribute to lung cancer (1,3). Histologically, lung cancer is broadly classified into small cell lung cancer (SCLC) and non-small cell lung cancer (NSCLC) (4). A total of ~80-85% lung cancers are NSCLC, encompassing various subtypes such as squamous cell carcinoma, adenocarcinoma, large cell carcinoma, adeno-squamous carcinoma and sarcomatoid carcinoma (4). Multiple genetic alterations have shown the potential to disrupt biological process or functionals, thereby facilitating the onset of lung cancer. Hence, the identification of susceptibility genes for lung cancer holds paramount clinical significance.

Cleft-lip and palate transmembrane protein-1-like (CLPTM1L) protein is identified as a protein associated with cisplatin resistance (5,6). The gene is situated on chromosome 5p15.33 and encodes a protein comprising 538 amino acids (6) with a molecular weight of 62 KDa (3,7). Functionally, the protein encoded by CLPTM1L is a membrane entity implicated in cisplatin resistance, where its overexpression in cisplatin-sensitive cells triggers apoptosis (8). Remarkably, both human and murine proteins exhibit 99% sequence identity, featuring 538 amino acids (5). Notably, within the chromosomal region 5p15, housing two genes, telomerase reverse transcriptase (TERT) and CLPTM1L, a susceptibility locus for lung cancer has been identified, with a potential role attributed to NKX2.4(9). Nonetheless, the interaction mechanism between these genes remains elusive. Moreover, CLPTM1L has been implicated in modulating inflammatory responses (10). Given the profound link between malignancy and heightened inflammation (11), numerous studies have scrutinized the potential of CLPTM1L mutation in precipitating tumorigenesis. Genome-wide association studies (GWAS) have pinpointed specific single nucleotide polymorphisms (SNPs) within the CLPTM1L gene (such as rs401681 and rs402710) as intricately intertwined with the onset and progression of various malignancies spanning lung cancer, breast cancer, pancreatic cancer, nasopharyngeal carcinoma and bladder cancer among others (3,7,12). In addition, clinical analyses have revealed that the expression levels of CLPTM1L in lung cancer tissue surpass those in healthy lung tissue, particularly evident in adenocarcinoma cells (5). Adenocarcinoma cells exhibit an average 2.15-fold elevation in CLPTM1L expression over normal immortalized cell lines (P = 3.59x10^-7^) (13). Since its initial identification of association with lung cancer susceptibility, numerous subsequent investigations have delved into the relationship between lung cancer risk and the rs401681 variant. Nevertheless, subsequent studies have yielded disparate findings (14-20). To comprehensively elucidate the association between lung cancer and rs401681, a meta-analysis encompassing correlational studies published prior to 2023 was undertaken.

## Materials and methods

### Literature search

A meticulous literature search was performed across multiple electronic databases, comprising PubMed (https://pubmed.ncbi.nlm.nih.gov/), Embase (https://www.embase.com), Cochrane Library (https://www.cochranelibrary.com/), Medline (https://www.nlm.nih.gov/medline/medline_overview.html), Google Scholar (https://scholar.google.com/), Wanfang (http://www.wanfangdata.com/) and Chinese National Knowledge Infrastructure (https://oversea.cnki.net/index/). Employing requisite combinations of keywords, such as ‘LC’ or ‘lung carcinoma’ or ‘lung neoplasm’ or ‘lung cancer’ and ‘rs401681 or CLPTM1L’, facilitated comprehensive retrieval. Moreover, additional relevant studies were identified through thorough examination of the reference lists of the included studies.

### Selection criteria

The inclusion criteria for eligible studies were as follows: i) Studies comprising both control and case groups; ii) studies investigating the association between CLPTM1L rs401681 and lung cancer susceptibility; iii) studies providing adequate data on genotype frequencies, or odds ratio (OR) with 95% confidence interval (95% CI) for lung cancer risk assessment and iv) investigations involving human subjects. A study that meets any of the following criteria was deemed to be non-compliant with the present meta-analysis: i) Comments, abstracts for conference, case reports, editorials or reviews; ii) insufficient data for OR calculation; iii) animal studies; and iv) studies lacking control groups.

### Quality assessment

The quality of all included studies was assessed utilizing the Newcastle-Ottawa Scale (NOS). The NOS assesses studies across three key dimensions: The selection of the study groups; the comparability of these groups; and the ascertainment of either the exposure or outcome of interest for case-control or cohort studies respectively. The scale yields a maximum score of 9(21). Finally, the studies with a total score surpassing 6 are considered high quality, those scoring between 4 and 6 are considered medium quality, and studies with a total score below 4 are categorized as low quality.

### Data extraction

Data extraction was independently carried out by two investigators. Any discordant opinion was solved through discussing to reach a final consensus. The data obtained from each included study encompassed the first author's name, publication year, region, ethnicity, disease type, sample size, genotype and/or allele frequencies of case and control subjects, and testimony of Hardy-Weinberg equilibrium in the control group.

### Statistical analysis

The statistical analyses were conducted using STATA software (version 17.0, StataCorp LP). The effect size of the correlation between the CLPTM1L rs401681 polymorphism and lung cancer risk was evaluated by pooled ORs with corresponding 95% CIs. χ^2^-based Q-test and I^2^ statistic were used to assess heterogeneity among studies. The random-effects model was used for the present meta-analysis. The significant heterogeneity was defined as P<0.05 for the Q test or I^2^>50%. Moreover, subgroup analysis was used to investigate potential sources of substantial heterogeneity. Publication bias was assessed by Egger's linear regression test. To test the robustness of the pooled outcomes, sensitivity analysis was employed using the leave-one-out method to identify the influence of individual studies on the overall effect estimate. P<0.05 was considered to indicate a statistically significant.

### In silico analysis

Aiming to elucidate the impact of polymorphisms on CLPTM1L, the authors embarked on an exploration of the association between the rs401681 and CLPTM1L expression levels via Genotype-Tissue Expression (GTEx) Analysis Release V6 (dbGaP Accession phs000424.v7.p2) data (22). To further investigate the potential influence of CLPTM1L expression on tumorigenesis, the authors endeavored to mine evidence from The Cancer Genome Atlas (TCGA) database (https://www.cancer.gov/ccg/research/genome-sequencing/tcga) by using the Kaplan-Meier. Additionally, the recently developed interactive platform, Gene Expression Profiling Interactive Analysis, was utilized to augment the inquiry (23-25).

## Results

### Characteristics of included studies

**Figure 1:**
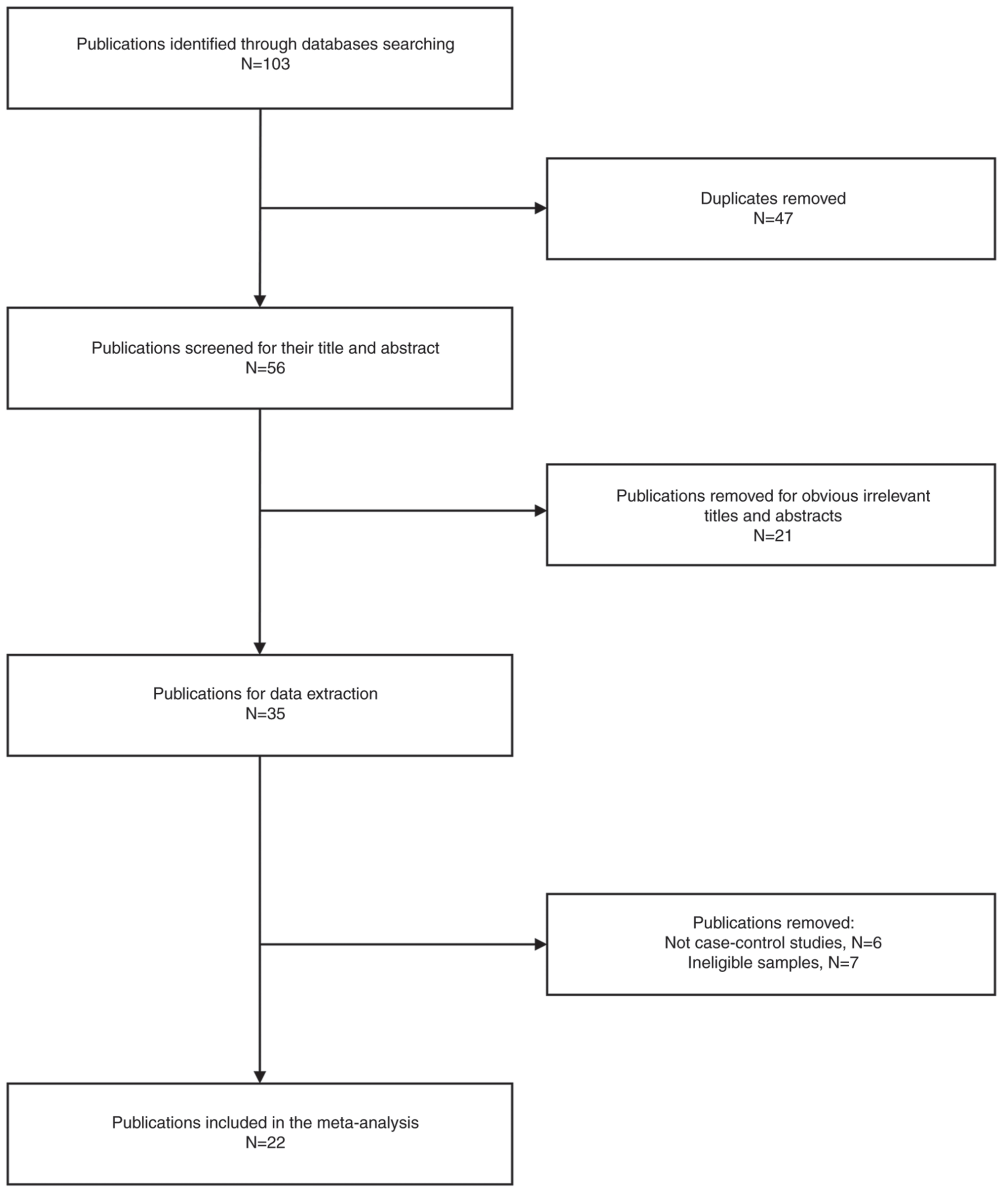
Flowchart for literature searching and selection.

Through the investigation of public databases, a total of 103 potential papers according to the search criteria were identified, from which 47 were subsequently excluded due to duplication (Fig. 1). Following this, a total of 22 studies were excluded due to unrelated topics. Moreover, 13 additional studies were excluded for lack of alignment with the case control design (n=6) and for including ineligible samples (n=7). Finally, 22 eligible articles were included (14,16,18,20,26-43). Among these studies, data pertaining to Caucasian populations were presented in 5 studies (26,29,30,38,42), whereas 17 studies included Asian populations. A more comprehensive overview of the studies included in the present meta-analysis is provided in Tables I and II.

### Susceptibility to lung cancer

The association between the CLPTM1L rs401681 and lung cancer is illustrated in Table III. OR<1 denotes reduced lung cancer risk associated with the exposed genotype compared with the non-exposed counterpart. The findings underscored a significant link between CLPTM1L rs401681 and altered lung cancer risk across all genetic comparisons. These comparisons encompass allele T vs. allele C (OR=0.93, 95% CI=0.88-0.99, P<0.001), TT + CT vs. CC (OR=0.91, 95%CI=0.87-0.96, P<0.001), TT vs. CC + CT (OR=0.88, 95% CI=0.80-0.96, P<0.001), TT vs. CC (OR=0.84, 95% CI=0.78-0.90, P<0.001), and CT vs. CC (OR=0.84, 95% CI=0.75-0.94, P=0.008).

### Heterogeneity and subgroup analysis

Q test and I^2^ findings revealed significant heterogeneity across four comparisons including allele T vs. allele C, TT+CT vs. CC, TT vs. CC, and CT vs. CC (Table III). Subgroup analysis results (Table III; Fig. S1, Fig. S2, Fig. S3, Fig. S4, Fig. S5, Fig. S6, Fig. S7, Fig. S8, Fig. S9 and Fig. S10) suggested that racial disparities may contribute to the considerable heterogeneity observed in the majority, if not all, of the comparisons. Upon stratification by ethnicity, a notable reduction in heterogeneity within the Caucasian subgroup was observed, although heterogeneity persisted within the Asian subgroup. Moreover, significant alterations in lung cancer risks were also detected in all genetic comparisons among Caucasians, whereas such alterations were statistically significant only in the TT vs. CC comparison within the Asian subgroup.

Furthermore, a total of 10 studies focusing on NSCLC and 3 studies focusing on SCLC furnished adequate data regarding genotype distribution, enabling the conduction of an additional subgroup analysis. The results showed that the association between rs401681 and the risks associated with NSCLC or SCLC did not attain statistical significance across all comparisons (Table III).

### Sensitivity analysis

From the results of sensitivity analysis in various comparisons (Fig. S11, Fig. S12, Fig. S13, Fig. S14 and Fig. S15), no substantial alterations in the re-obtained ORs were observed when compared with the initial ORs. This indicated the robustness and consistency of these findings.

### Publication bias

The P-value of Egger's linear regression test (Table III) indicated the absence of noteworthy publication bias across all comparisons.

### In silico analysis

**Figure 2:**
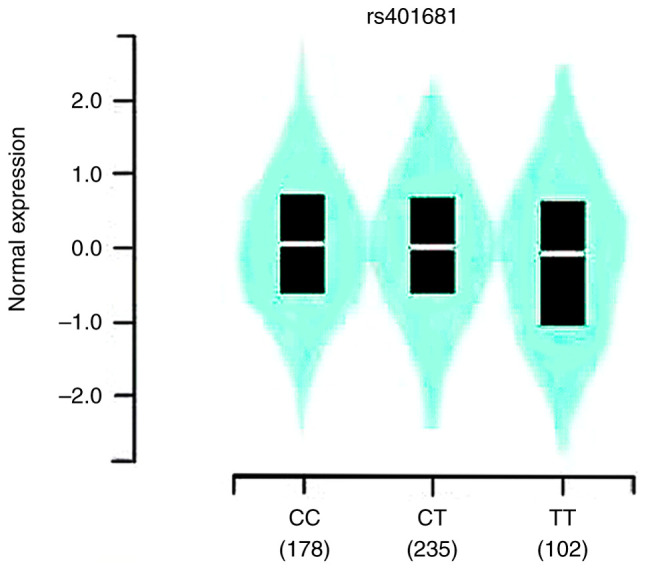
*In silico* analysis of CLPTM1L expression concerned to rs401681 and lung cancer. CLPTM1L mRNA expression by expression quantitative trait loci analysis in human tissues based on Genotype-Tissue Expression database. CLPTM1L, cleft-lip and palate transmembrane protein-1-like.

**Figure 3:**
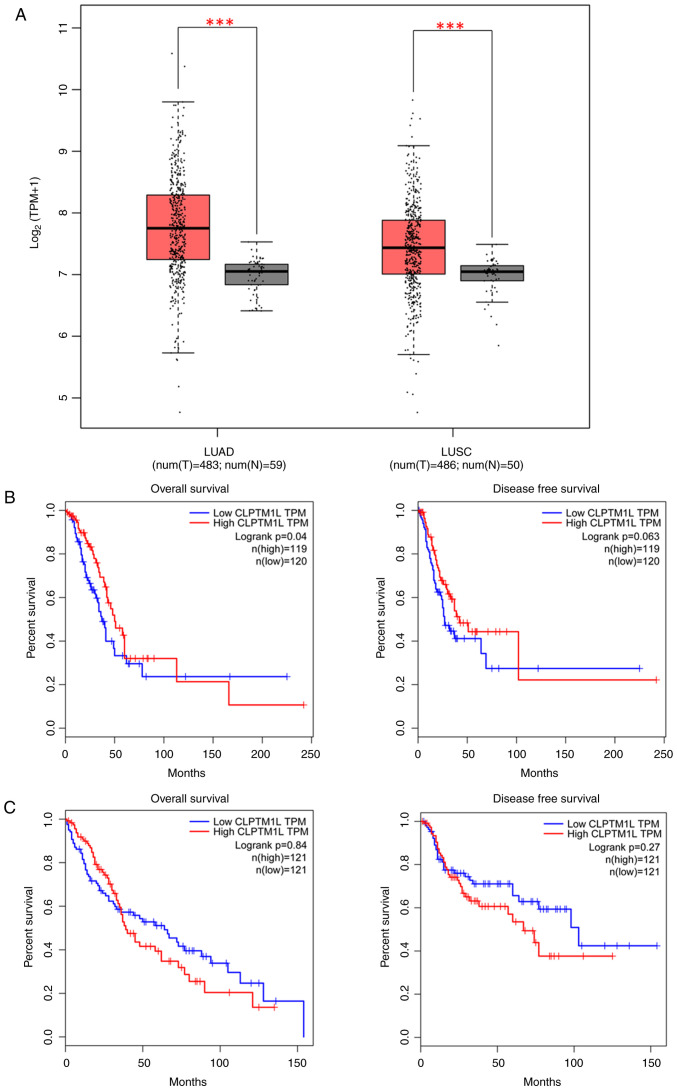
*In silico* analysis of CLPTM1L expression. (A) Comparison of CLPTM1L expression between tumor site and matched normal tissue in The Cancer Genome Atlas database. (B) The association between the expression of CLPTM1L and the overall survival time and disease-free survival time of LUAD. (C) The association between the expression of CLPTM1L and the overall survival time, disease free survival time of LUSC. CLPTM1L, cleft-lip and palate transmembrane protein-1-like; LUAD, lung adenocarcinoma; LUSC, lung squamous cell carcinoma. ^***^P<0.001.

Based on the GTEx portal data, the analysis revealed that the mutant allele was associated with increased expression of CLPTM1L mRNA at rs401681 (P=0.02) (Fig. 2). Evidence gleaned from TCGA database further underscored this finding, demonstrating elevated CLPTM1L expression levels in lung adenocarcinoma (LUAD) tissue compared with normal tissue [CLPTM1L expression=(5.5x10^3^) vs. (2.8x10^3^), P<0.001], as well as in lung squamous cell carcinoma (LUSC) [CLPTM1L expression=(4.6x10^3^) vs. (2.9x10^3^), P<0.001] (Fig. 3A).

Moreover, whether the expression level of CLPTM1 influences the disease-free survival (DFS) and overall survival (OS) of lung cancer was investigated. The Kaplan-Meier estimations revealed a notable discrepancy in OS between the low and high CLPTM1 transcripts per kilobase million (TPM) groups in LUAD (log-rank P=0.04), albeit a difference in DFS close to statistical significance (log-rank P=0.063). On the other hand, no distinction was observed between high and low CLPTM1 TPM groups in LUSC (log-rank P=0.84 for OS and 0.27 for DFS). Details are presented in Fig. 3B and C.

## Discussion

In the present study, the impact of CLPTM1L rs401681 within the locus 5p15.33 on lung cancer incidence was scrutinized, and it was found that it had a remarkable association with onset of lung cancer. Prior investigations yield a discordant landscape regarding the role of CLPTM1L rs401681 in the occurrence of lung cancer. There was no overall correlation between CLPTM1L rs401681 and the risk of lung cancer in several previous studies (20,28), whereas others reported that allele T of CLPTM1L rs401681 decreases the susceptibility of lung cancer (33,39,40,43). A precedent meta-analysis hinted at an association between CLPTM1L rs401681 and lung cancer (14), albeit with a caveat on sample size constraints acknowledged by the authors. Subsequently, emerging studies exploring the relationship between CLPTM1L rs401681 and lung cancer risk have been published (16,18,20,32,34,39,41,44,45). To further determine the relationship between CLPTM1L rs401681 polymorphism and lung cancer risk, the authors embarked on a meta-analysis of all the literature published before 2023. In the present study, the association between CLPTM1L rs401681 polymorphism and lung cancer risk was systematically reviewed and was based on ten case-control studies, encompassing 14,195 cases and 16,732 controls. The meta-analysis demonstrated that CLPTM1L rs401681 polymorphism was associated with the occurrence of lung cancer in all the genetic comparisons investigated, suggestive of a protective effect conferred by the T allele of CLPTM1L rs401681 mutation against lung cancer. Nevertheless, the I^2^ statistic and statistical Q test revealed significant heterogeneity, with ethnic origin emerging as a plausible primary contributor to this observed heterogeneity.

Upon stratification by race, a statistically significant association of CLPTM1L rs401681 with lung cancer risk was observed in each genetic comparison among Caucasians, whereas it was only notable for the TT vs. CC comparison among Asians. Although the exact cause for racial disparities is not entirely clear, one possible cause may lie within the genetic context. When considering subgroups based on histology, it was found that the association between rs401681 and NSCLC or SCLC risks was not statistically significant for all comparisons. This suggested that CLPTM1L rs401681 variant genotypes may not exert an effect on the pathological differentiation of lung cancer. Future investigations should aim to elucidate the underlying mechanisms, taking into account factors such as histology or race heterogeneity, to provide a more nuanced understanding of the impact of CLPTM1L gene polymorphism on lung cancer risk and to tailor personal therapy.

The association between lung cancer and the CLPTM1L rs401681 polymorphism may be confounded by other influential SNPs, particularly those situated within TERT-CLPTM1L region. This is especially pertinent for SNPs in close linkage disequilibrium with rs401681, such as rs31490 and rs414965, or those directly implicated in lung cancer. In addition, variations in matching criteria, selection bias, and limited data availability in the present study may lead to statistical insufficiency to identify subtle differences, and a fluctuated risk estimate. Several findings underscored the critical importance of assessing genetic influences across diverse populations for disease onset and progression.

The present study possessed several limitations. Primarily, the intricate interplay between gene-gene and gene-environment interactions in lung cancer risk poses challenges for evaluating genotype polymorphism alongside potential psychological and environmental factors. Consequently, the assessment primarily focused on delineating the risk coefficient associated with aberrant genotypes. Secondly, the present study only included Chinese and English studies, potentially introducing unacknowledged language bias. Thirdly, the possibility of chance findings cannot be entirely discounted, given the limited patient cohorts within the present stratified analyses. These subgroup associations necessitate validation within larger cohorts for enhanced precision. Lastly, due to lacking certain subgroup data of included studies, some important subgroups such as sex are not analyzed in the present meta-analysis. In future research, investigators should focus more on this important issue.

In conclusion, the current meta-analysis demonstrates a significant association between the CLPTM1L rs401681 polymorphism and the risk of lung cancer, wherein the T allele exhibits a protective effect. These findings offer preliminary insights into assessing the risk of lung cancer based on the SNP rs401681 genotype, thus laying the groundwork for personalized medicine endeavors.

## Supplementary Material

Forest plot of the relationship between the cleft-lip and palate transmembrane protein-1-like rs401681 polymorphism and lung susceptibility (including racial subgroup analysis) in the allele T vs. allele C. The squares and horizontal lines correspond to the study-specific OR and 95% CI. The area of a square reflects the weight (reciprocal of variance). The diamond represents the total OR and 95% CI. OR, odds ratio; CI, confidence interval.

Forest plot of the relationship between the cleft-lip and palate transmembrane protein-1-like rs401681 polymorphism and lung susceptibility for racial subgroup analysis in the allele T vs. allele C. The squares and horizontal lines correspond to the study-specific OR and 95% CI. The area of a square reflects the weight (reciprocal of variance). The diamond represents the summary OR and 95% CI. OR, odds ratio; CI, confidence interval.

Forest plot of the relationship between the cleft-lip and palate transmembrane protein-1-like rs401681 polymorphism and lung susceptibility (including racial subgroup analysis) in the TT + CT vs. CC. The squares and horizontal lines correspond to the study-specific OR and 95% CI. The area of a square reflects the weight (reciprocal of variance). The diamond represents the summary OR and 95% CI. OR, odds ratio; CI, confidence interval.

Forest plot of the relationship between the cleft-lip and palate transmembrane protein-1-like rs401681 polymorphism and lung susceptibility for racial subgroup analysis in the TT + CT vs. CC. The squares and horizontal lines correspond to the study-specific OR and 95% CI. The area of a square reflects the weight (reciprocal of variance). The diamond represents the summary OR and 95% CI. OR, odds ratio; CI, confidence interval.

Forest plot of the relationship between the cleft-lip and palate transmembrane protein-1-like rs401681 polymorphism and lung susceptibility (including racial subgroup analysis) in the TT vs. CC + CT. The squares and horizontal lines correspond to the study-specific OR and 95% CI. The area of a square reflects the weight (reciprocal of variance). The diamond represents the summary OR and 95% CI. OR, odds ratio; CI, confidence interval.

Forest plot of the relationship between the cleft-lip and palate transmembrane protein-1-like rs401681 polymorphism and lung susceptibility for racial subgroup analysis in the TT vs. CC + CT. The squares and horizontal lines correspond to the study-specific OR and 95% CI. The area of a square reflects the weight (reciprocal of variance). The diamond represents the summary OR and 95% CI. OR, odds ratio; CI, confidence interval.

Forest plot of the relationship between the cleft-lip and palate transmembrane protein-1-like rs401681 polymorphism and lung susceptibility (including racial subgroup analysis) in the TT vs. CC. The squares and horizontal lines correspond to the study-specific OR and 95% CI. The area of a square reflects the weight (reciprocal of variance). The diamond represents the summary OR and 95% CI. OR, odds ratio; CI, confidence interval.

Forest plot of the relationship between the cleft-lip and palate transmembrane protein-1-like rs401681 polymorphism and lung susceptibility for racial subgroup analysis in the allele TT vs. CC. The squares and horizontal lines correspond to the study-specific OR and 95% CI. The area of a square reflects the weight (reciprocal of variance). The diamond represents the summary OR and 95% CI. OR, odds ratio; CI, confidence interval.

Forest plot of the relationship between the cleft-lip and palate transmembrane protein-1-like rs401681 polymorphism and lung susceptibility (including racial subgroup analysis) in the CT vs. CC. The squares and horizontal lines correspond to the study-specific OR and 95% CI. The area of a square reflects the weight (reciprocal of variance). The diamond represents the summary OR and 95% CI. OR, odds ratio; CI, confidence interval.

Forest plot of the relationship between the cleft-lip and palate transmembrane protein-1-like rs401681 polymorphism and lung susceptibility for racial subgroup analysis in the CT vs. CC. The squares and horizontal lines correspond to the study-specific OR and 95% CI. The area of a square reflects the weight (reciprocal of variance). The diamond represents the summary OR and 95% CI. OR, odds ratio; CI, confidence interval.

Sensitivity analysis for testing the stability of overall estimates of allele T vs. allele C.

Sensitivity analysis for testing the stability of overall estimates of TT + CT vs. CC.

Sensitivity analysis for testing the stability of overall estimates of TT vs. CC + CT.

Sensitivity analysis for testing the stability of overall estimates of TT vs. CC.

Sensitivity analysis for testing the stability of the overall estimate in the CT vs. CC.

## Figures and Tables

**Table I:** Main characteristics of the included studies in the present meta-analysis.

		Sample size		Case genotype			Control genotype					
First author, year	Race	Case	Control	CC	CT	TT	CC	CT	TT	NOS score	HWE	(Refs.)
Wang *et al*, 2008	Caucasian	2,396	3,051	868	1,134	394	994	1,506	551	7	Y	(38)
Amos *et al*, 2008	Caucasian	1,153	1,137	396	570	187	336	596	205	8	Y	(26)
Hung *et al*, 2008	Caucasian	1,920	2,517	710	949	261	857	1,239	421	8	Y	(30)
Zienolddiny *et al*, 2009	Caucasian	341	431	107	177	57	117	224	90	8	Y	(42)
Yoon *et al*, 2010	Asian	431	341	117	224	90	107	177	57	8	Y	(40)
Bae *et al*, 2012	Asian	1,086	1,079	545	434	107	499	484	96	7	Y	(27)
Chen *et al*, 2012	Asian	195	228	95	90	10	126	77	25	7	Y	(28)
Li *et al*, 2013	Asian	464	536	218	205	41	244	234	58	8	Y	(14)
Myneni *et al*, 2013	Asian	350	441	181	135	34	194	200	47	8	Y	(35)
Wang *et al*, 2013	Asian	492	486	245	201	46	215	203	68	8	Y	(37)
de Mello *et al*, 2013	Caucasian	144	144	40	77	27	44	75	25	8	Y	(29)
Jiang *et al*, 2013	Asian	726	860	371	289	66	395	378	87	8	Y	(31)
Ke *et al*, 2013	Asian	611	1,062	324	231	56	495	459	108	8	Y	(33)
Sun *et al*, 2013	Asian	400	200	186	188	26	95	86	19	8	Y	(36)
Lv *et al*, 2013	Asian	602	1,060	315	231	56	493	459	108	8	Y	(43)
Zhao *et al*, 2014	Asian	951	954	436	424	91	437	424	93	8	Y	(20)
Xun *et al*, 2014	Asian	228	299	112	102	14	132	132	35	8	Y	(18)
Zhang *et al*, 2014	Asian	366	364	182	149	35	167	169	28	8	Y	(41)
Liang *et al*, 2014	Asian	309	308	145	139	25	137	134	37	8	Y	(16)
Liu *et al*, 2015	Asian	292	319	157	112	23	138	146	35	8	Y	(34)
Jin *et al*, 2016	Asian	554	695	276	234	44	308	302	85	8	Y	(32)
Xiao *et al*, 2017	Asian	199	220	85	99	15	128	79	13	8	Y	(39)

NOS, Newcastle-Ottawa Scale; HWE, Hardy-Weinberg equilibrium; Y, conform to HWE.

**Table II:** Comparative analysis of genotype frequencies and the risk of lung cancer in the subgroups.

			Sample size		Case genotype			Control genotype			
First author, year	Race	Histology	Case	Control	CC	CT	TT	CC	CT	TT	(Refs.)
Zienolddiny *et al*, 2009	Caucasian	NSCLC	341	431	107	177	57	117	224	90	(42)
Yoon *et al*, 2010	Asian	NSCLC	431	341	117	224	90	107	177	57	(40)
Chen *et al*, 2012	Asian	SCLC	16	228	8	8	0	126	77	25	(28)
Chen *et al*, 2012	Asian	NSCLC	180	228	87	82	10	126	77	25	(28)
Li *et al*, 2013	Asian	NSCLC	464	536	218	205	41	244	234	58	(14)
Wang *et al*, 2013	Asian	SCLC	99	486	45	45	9	215	203	68	(37)
Wang *et al*, 2013	Asian	NSCLC	393	486	200	156	37	215	203	68	(37)
de Mello *et al*, 2013	Caucasian	NSCLC	144	144	40	77	27	44	75	25	(29)
Ke *et al*, 2013	Asian	NSCLC	427	1,062	229	155	37	493	459	108	(33)
Sun *et al*, 2013	Asian	NSCLC	400	200	186	188	26	95	86	19	(36)
Zhao *et al*, 2014	Asian	SCLC	703	954	334	301	68	437	424	93	(20)
Zhao *et al*, 2014	Asian	NSCLC	139	954	59	68	12	437	424	93	(20)
Zhang *et al*, 2014	Asian	NSCLC	366	364	182	149	35	167	169	28	(41)

NSCLC, non-small cell lung cancer; SCLC, small cell lung cancer.

**Table III:** Result of meta-analysis for CLPTM1L rs401681 polymorphism and lung cancer risk.

			T vs. C			TT + CT vs. CC			TT vs. CC + CT			TT vs. CC			CT vs. CC		
Variables	N	Case/control	OR (95% CI)	P_h_/I^2^(%)/Pz	P_E_	OR (95% CI)	P_h_/I^2^(%)/Pz	P_E_	OR (95% CI)	P_h_/I^2^(%)/Pz	P_E_	OR (95% CI)	P_h_/I^2^(%)/Pz	P_E_	OR (95% CI)	P_h_/I^2^(%)/Pz	P_E_
Total	22	14195/16732	0.93 (0.88-0.99)	0.000/60.9/0.000	0.406	0.91 (0.87-0.96)	0.000/63.7/0.000	0.229	0.88 (0.80-0.96)	0.054/35/0.000	0.617	0.84 (0.78-0.90)	0.008/46.9/0.000	0.886	0.84 (0.75-0.94)	0.000/46.9/0.008	0.162
Ethnicity																	
Caucasian	5	5954/7280	0.89 (0.85-0.93)	0.756/0/0.000	/	0.86 (0.80-0.92)	0.722/0/0.000	/	0.85 (0.78-0.93)	/	/	0.79 (0.71-0.87)	0.688/0/0.000		0.79 (0.71-0.91)	0.688/0/0.000	/
Asian	17	8241/9452	0.95 (0.88-1.03)	0.000/66.2/0.046	/	0.96 (0.90-1.02)	0.000/68.2/0.151	/	0.88 (0.76-1.01)	/	/	0.89 (0.80-0.99)	0.005/53.8/0.069		0.86 (0.73-1.01)	0.005/53.8/0.737	/
Histology																	
NSCLC	10	3849/4746	0.93 (0.85-1.01)	0.123/35.7/0.087	/	0.92 (0.82-1.04)	0.114/36.8/0.178	/	0.88 (0.74-1.05)	0.131/34.6/0.154	/	0.85 (0.70-1.03)	0.121/35.9/0.099		0.95 (0.84-1.07)	0.096/39.3/0.383	/
SCLC	3	254/1668	0.93 (0.87-1.00)	0.241/20.1/0.756	/	0.94 (0.85-1.04)	0.189/25.3/0.601	/	0.87 (0.74-1.01)	0.214/22.7/0.190	/	0.84 (0.72-1.00)	0.227/21.4/0.318		0.97 (0.87-1.09)	0.124/32.3/0.280	/

Random-effect model was used. N, number of comparisons; Ph, P-value of Q test for heterogeneity; PZ, P-value of Z test; Pe, P-value of Egger's test; NSCLC, non-small cell lung cancer; SCLC, small cell lung cancer.

## Data Availability

The data generated in the present study are included in the figures and/or tables of this article.
