# Supplementary material for: The updated relationship between the cleft‑lip and palate transmembrane protein‑1‑like rs401681 and lung cancer risk: A systematic review and meta‑analysis
Source: Mol Clin Oncol. 2024 Jul 31;21(4):70. doi: 10.3892/mco.2024.2768 (PMC11304168; doi:10.3892/mco.2024.2768)

Figure S1. Forest plot of the relationship between the cleft-lip and palate transmembrane protein-1-like rs401681 polymorphism and lung susceptibility (including racial subgroup analysis) in the allele T vs. allele C. The squares and horizontal lines correspond to the study-specific OR and 95% CI. The area of a square reflects the weight (reciprocal of variance). The diamond represents the total OR and 95% CI. OR, odds ratio; CI, confidence interval.

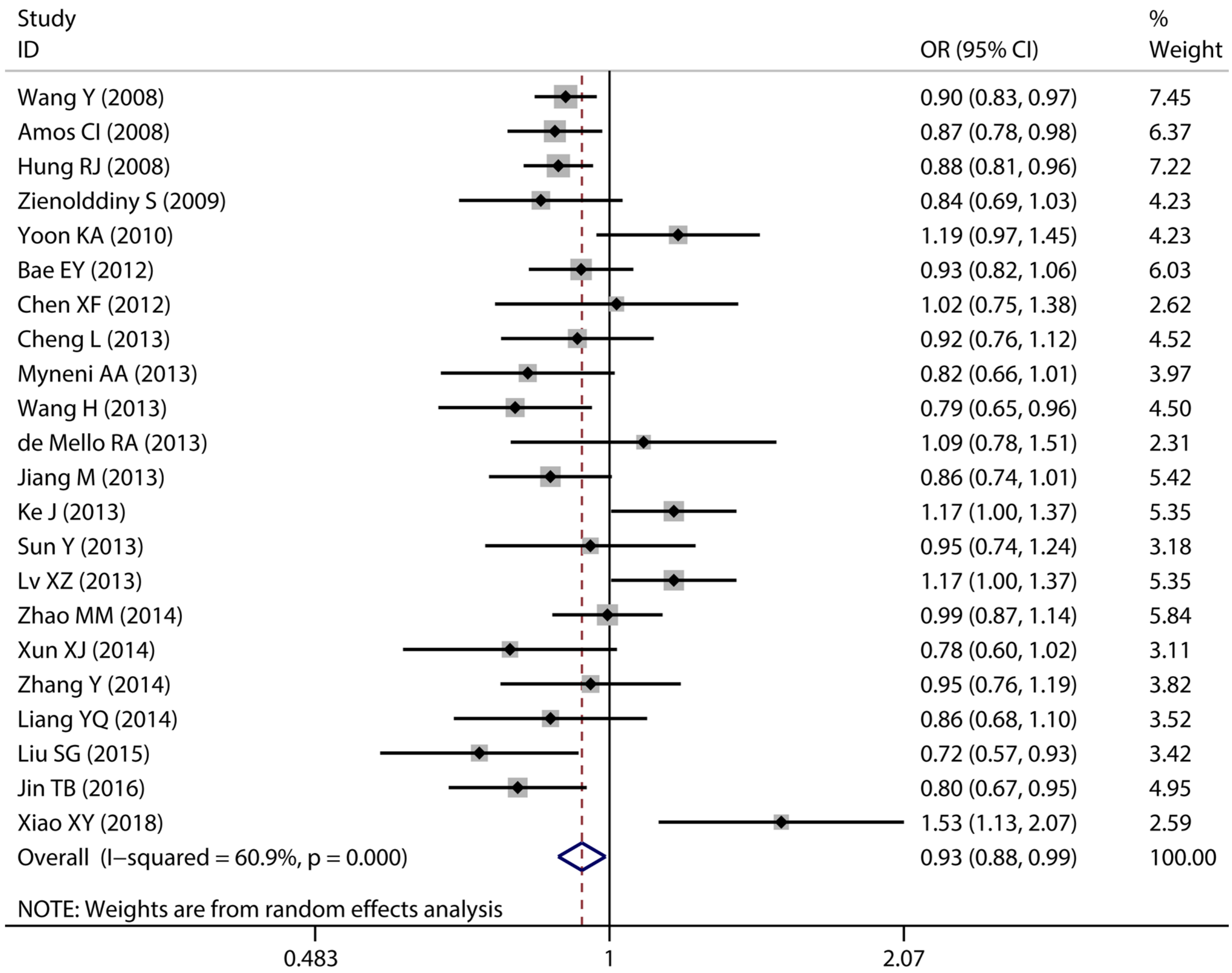

Figure S2. Forest plot of the relationship between the cleft-lip and palate transmembrane protein-1-like rs401681 polymorphism and lung susceptibility for racial subgroup analysis in the allele T vs. allele C. The squares and horizontal lines correspond to the study-specific OR and 95% CI. The area of a square reflects the weight (reciprocal of variance). The diamond represents the summary OR and 95% CI. OR, odds ratio; CI, confidence interval.

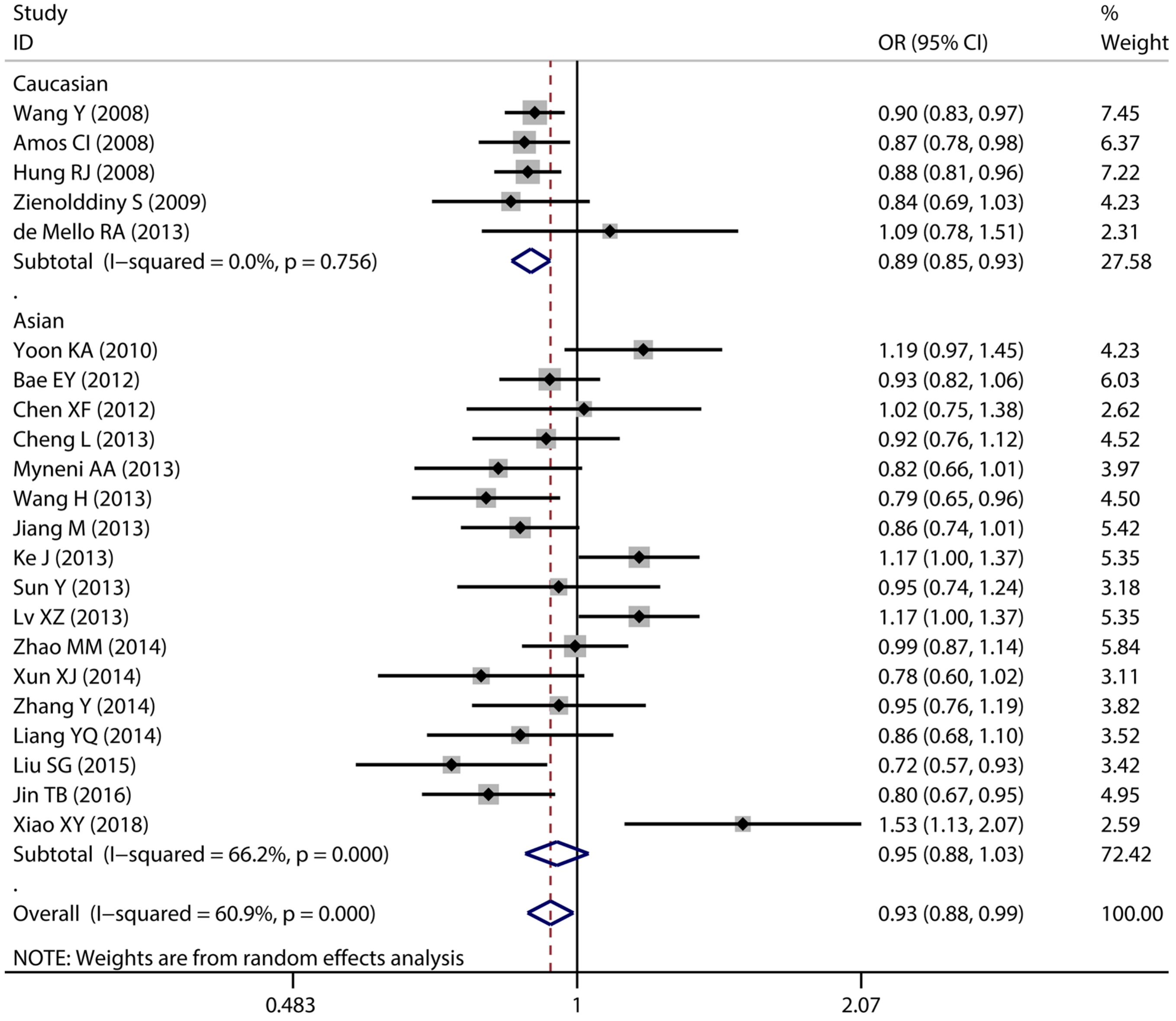

Figure S3. Forest plot of the relationship between the cleft-lip and palate transmembrane protein-1-like rs401681 polymorphism and lung susceptibility (including racial subgroup analysis) in the TT + CT vs. CC. The squares and horizontal lines correspond to the study-specific OR and 95% CI. The area of a square reflects the weight (reciprocal of variance). The diamond represents the summary OR and 95% CI. OR, odds ratio; CI, confidence interval.

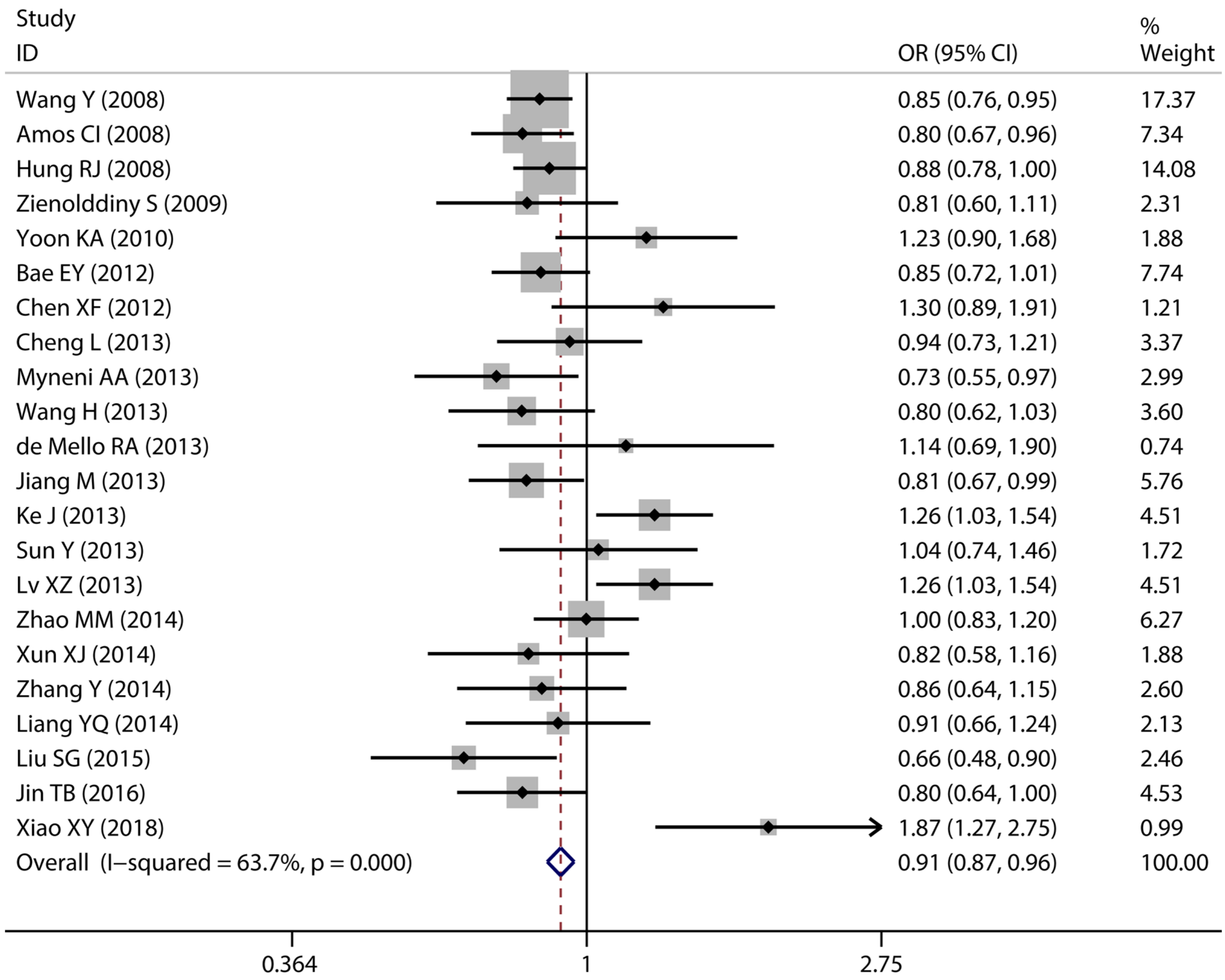

Figure S4. Forest plot of the relationship between the cleft-lip and palate transmembrane protein-1-like rs401681 polymorphism and lung susceptibility for racial subgroup analysis in the TT + CT vs. CC. The squares and horizontal lines correspond to the study-specific OR and 95% CI. The area of a square reflects the weight (reciprocal of variance). The diamond represents the summary OR and 95% CI. OR, odds ratio; CI, confidence interval.

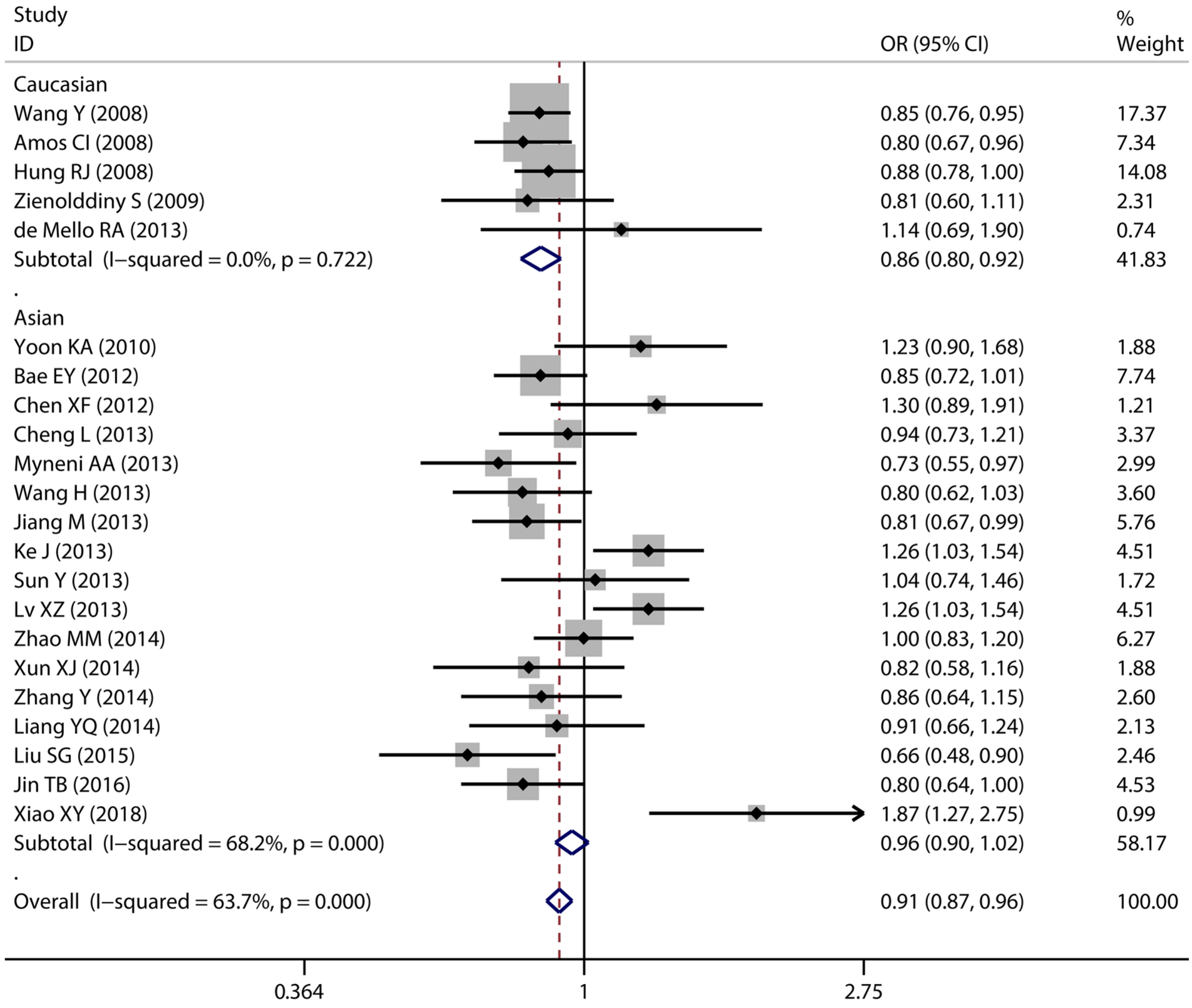

Figure S5. Forest plot of the relationship between the cleft-lip and palate transmembrane protein-1-like rs401681 polymorphism and lung susceptibility (including racial subgroup analysis) in the TT vs. CC + CT. The squares and horizontal lines correspond to the study-specific OR and 95% CI. The area of a square reflects the weight (reciprocal of variance). The diamond represents the summary OR and 95% CI. OR, odds ratio; CI, confidence interval.

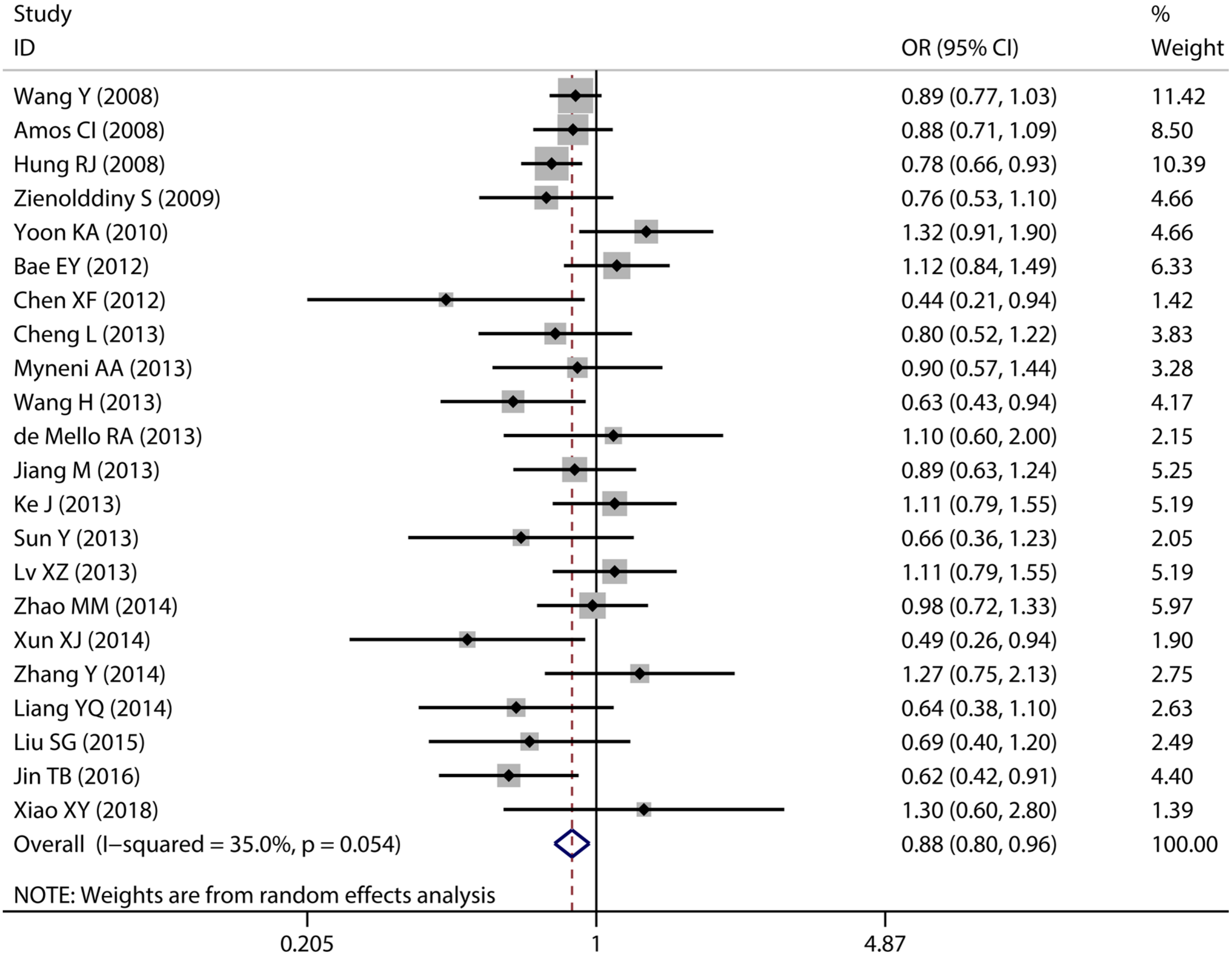

Figure S6. Forest plot of the relationship between the cleft-lip and palate transmembrane protein-1-like rs401681 polymorphism and lung susceptibility for racial subgroup analysis in the TT vs. CC + CT. The squares and horizontal lines correspond to the study-specific OR and 95% CI. The area of a square reflects the weight (reciprocal of variance). The diamond represents the summary OR and 95% CI. OR, odds ratio; CI, confidence interval.

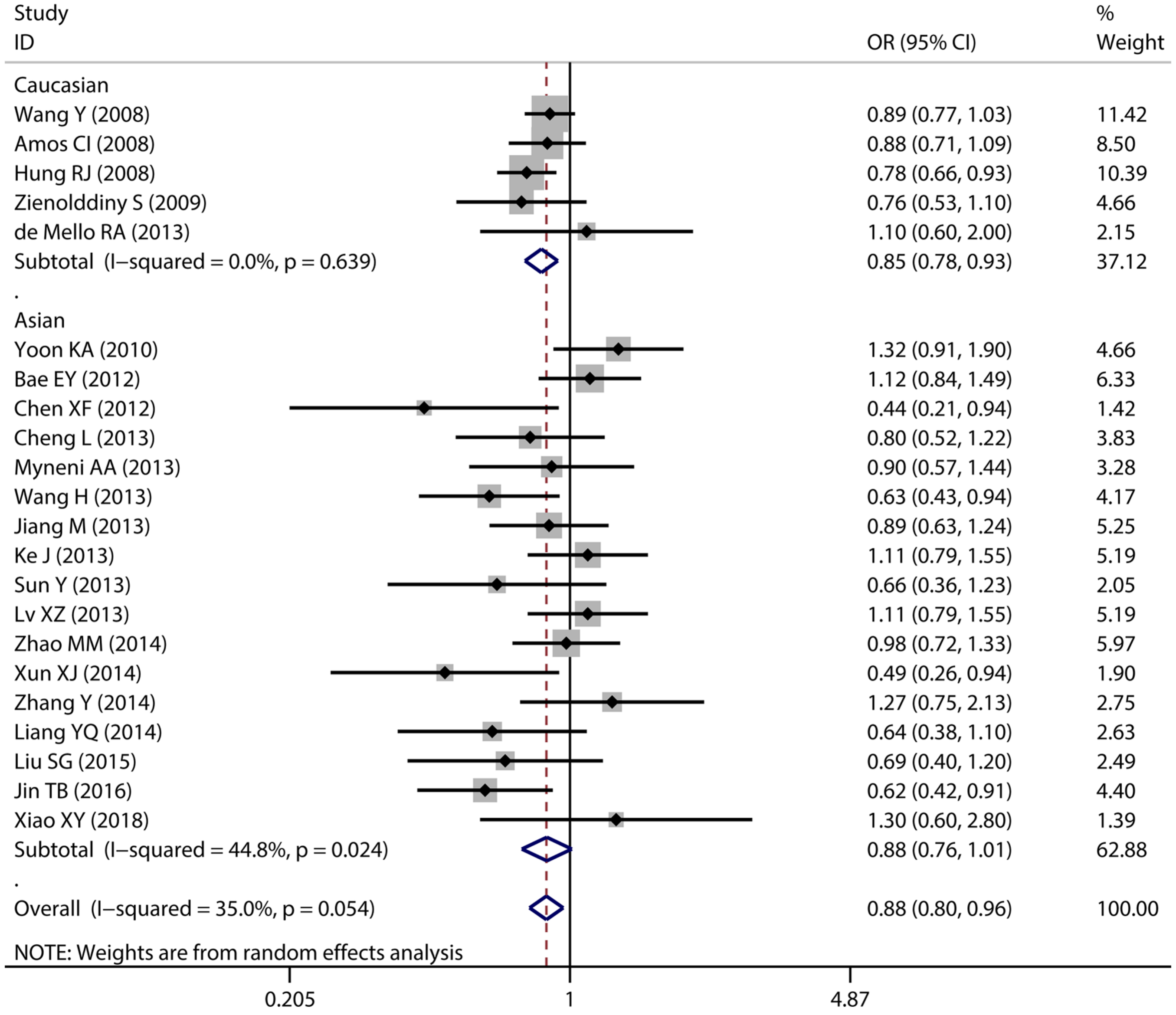

Figure S7. Forest plot of the relationship between the cleft-lip and palate transmembrane protein-1-like rs401681 polymorphism and lung susceptibility (including racial subgroup analysis) in the TT vs. CC. The squares and horizontal lines correspond to the study-specific OR and 95% CI. The area of a square reflects the weight (reciprocal of variance). The diamond represents the summary OR and 95% CI. OR, odds ratio; CI, confidence interval.

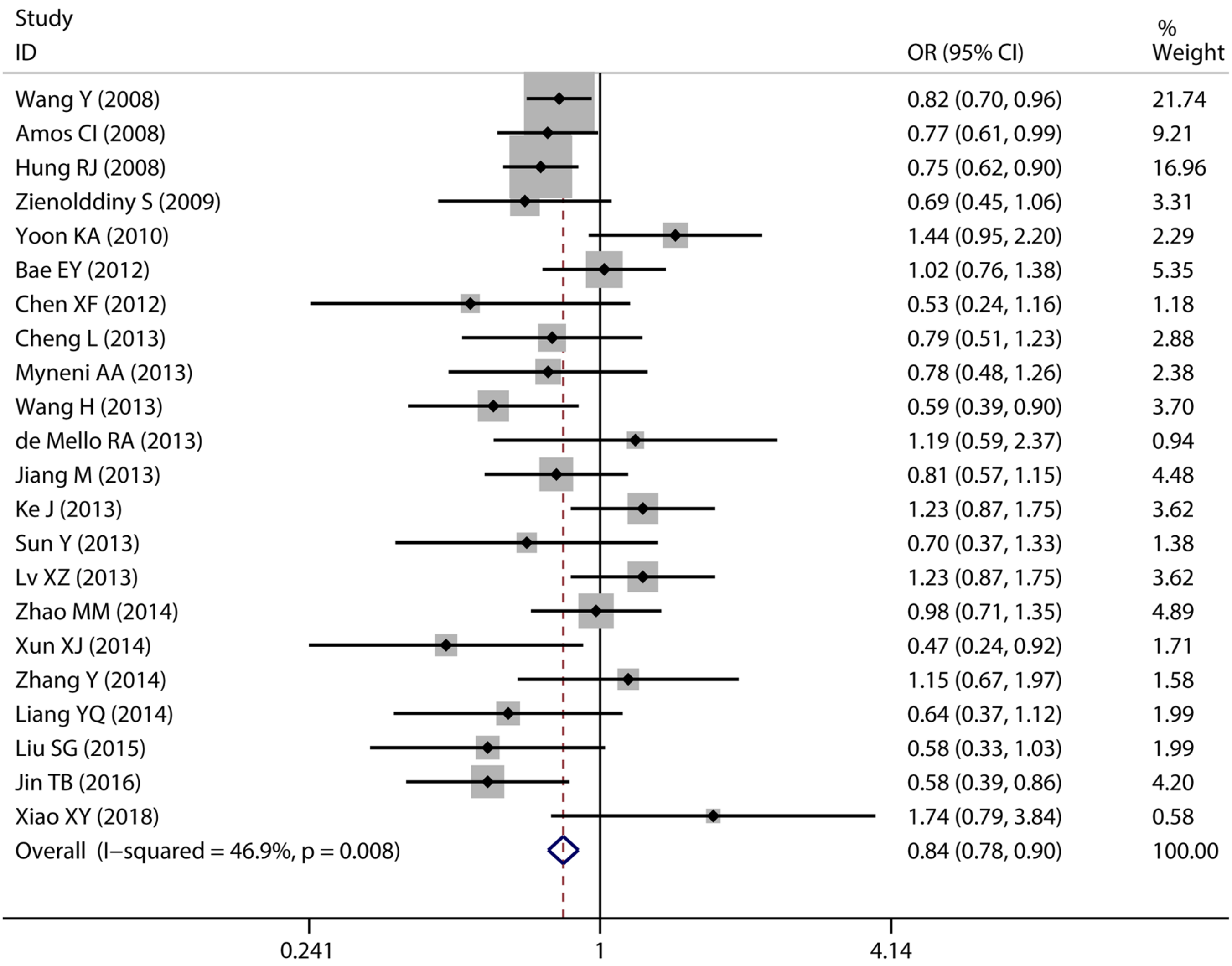

Figure S8. Forest plot of the relationship between the cleft-lip and palate transmembrane protein-1-like rs401681 polymorphism and lung susceptibility for racial subgroup analysis in the allele TT vs. CC. The squares and horizontal lines correspond to the study-specific OR and 95% CI. The area of a square reflects the weight (reciprocal of variance). The diamond represents the summary OR and 95% CI. OR, odds ratio; CI, confidence interval.

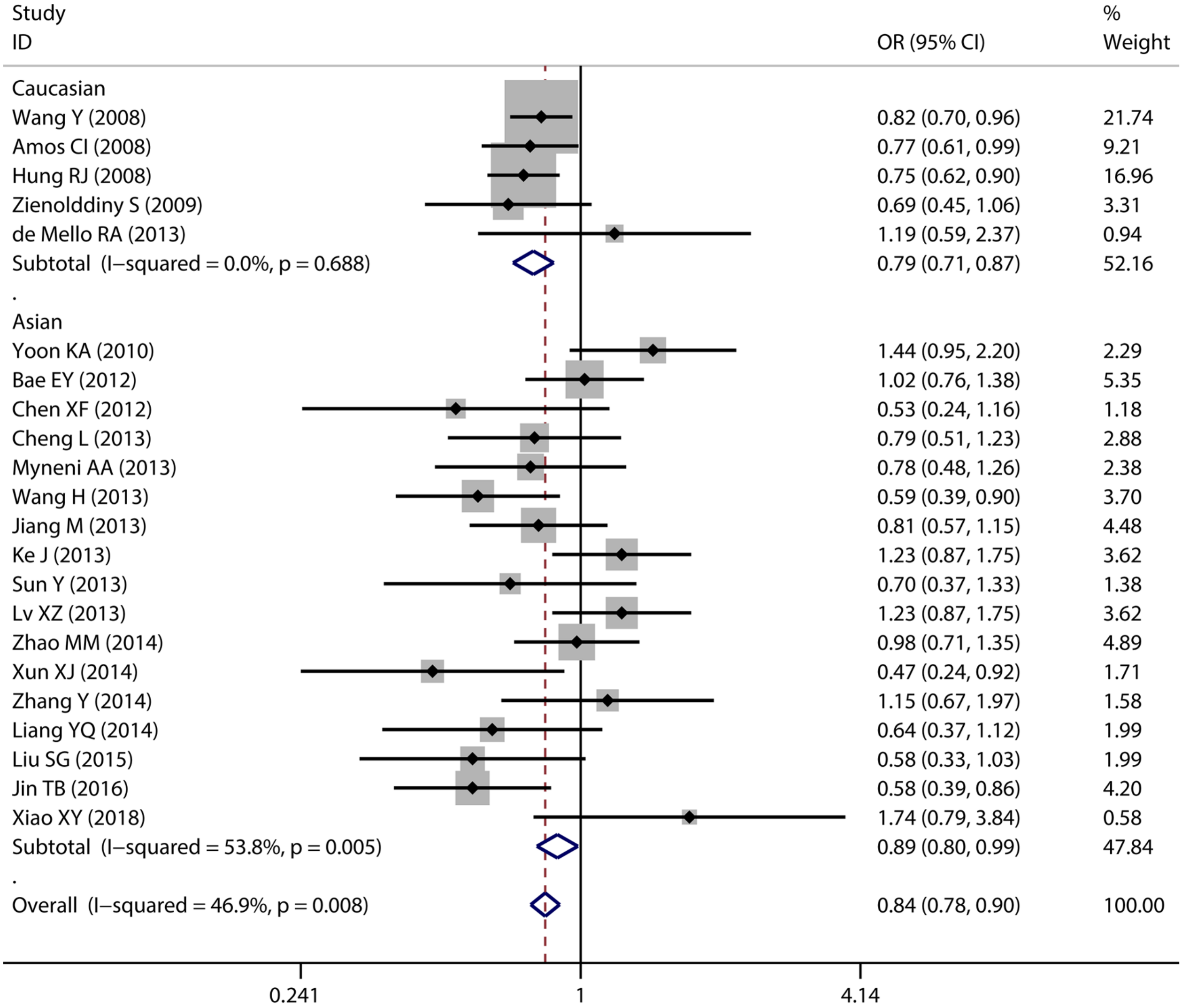

Figure S9. Forest plot of the relationship between the cleft-lip and palate transmembrane protein-1-like rs401681 polymorphism and lung susceptibility (including racial subgroup analysis) in the CT vs. CC. The squares and horizontal lines correspond to the study-specific OR and 95% CI. The area of a square reflects the weight (reciprocal of variance). The diamond represents the summary OR and 95% CI. OR, odds ratio; CI, confidence interval.

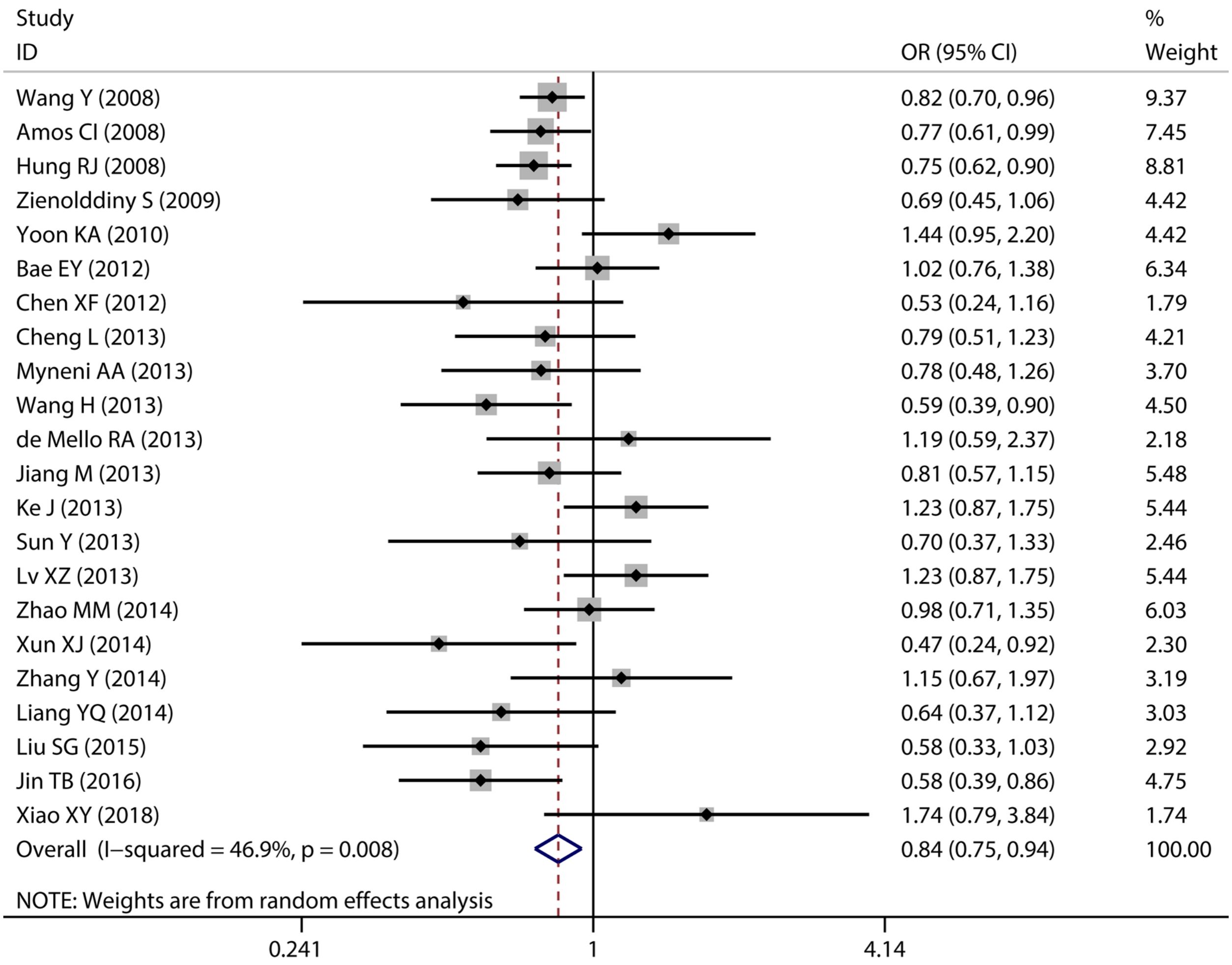

Figure S10. Forest plot of the relationship between the cleft-lip and palate transmembrane protein-1-like rs401681 polymorphism and lung susceptibility for racial subgroup analysis in the CT vs. CC. The squares and horizontal lines correspond to the study-specific OR and 95% CI. The area of a square reflects the weight (reciprocal of variance). The diamond represents the summary OR and 95% CI. OR, odds ratio; CI, confidence interval.

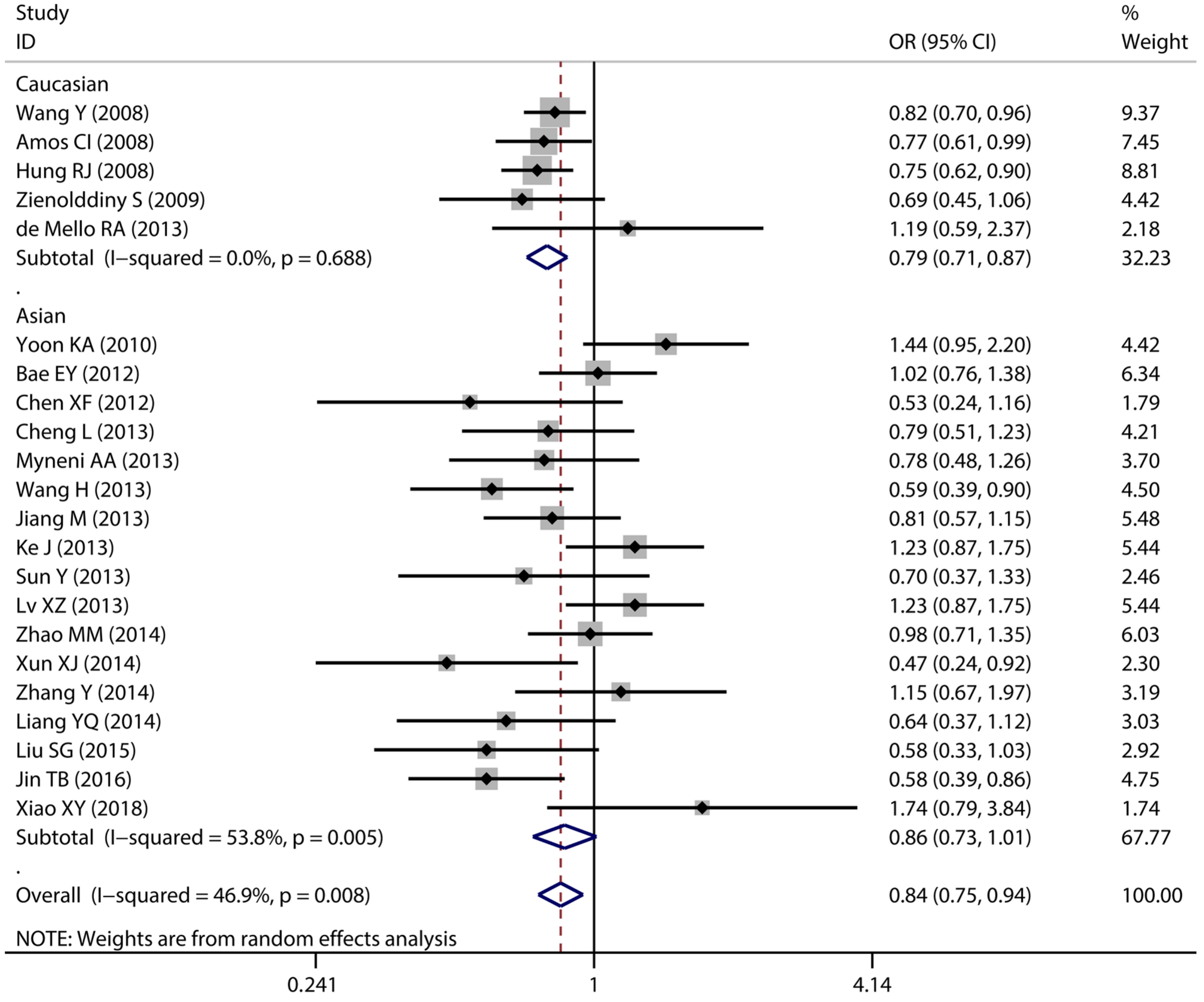

Figure S11. Sensitivity analysis for testing the stability of overall estimates of allele T vs. allele C.

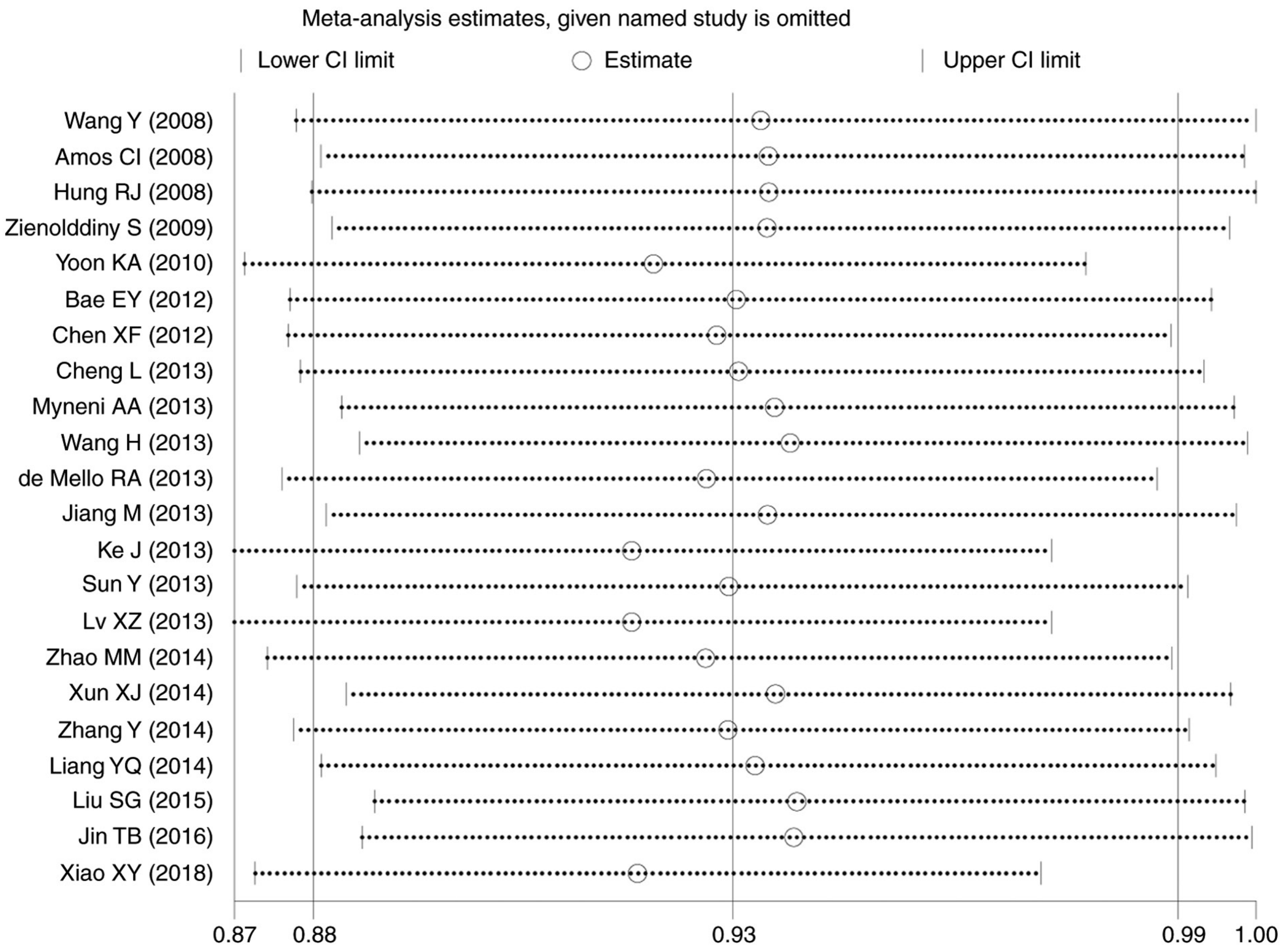

Figure S12. Sensitivity analysis for testing the stability of overall estimates of TT + CT vs. CC.

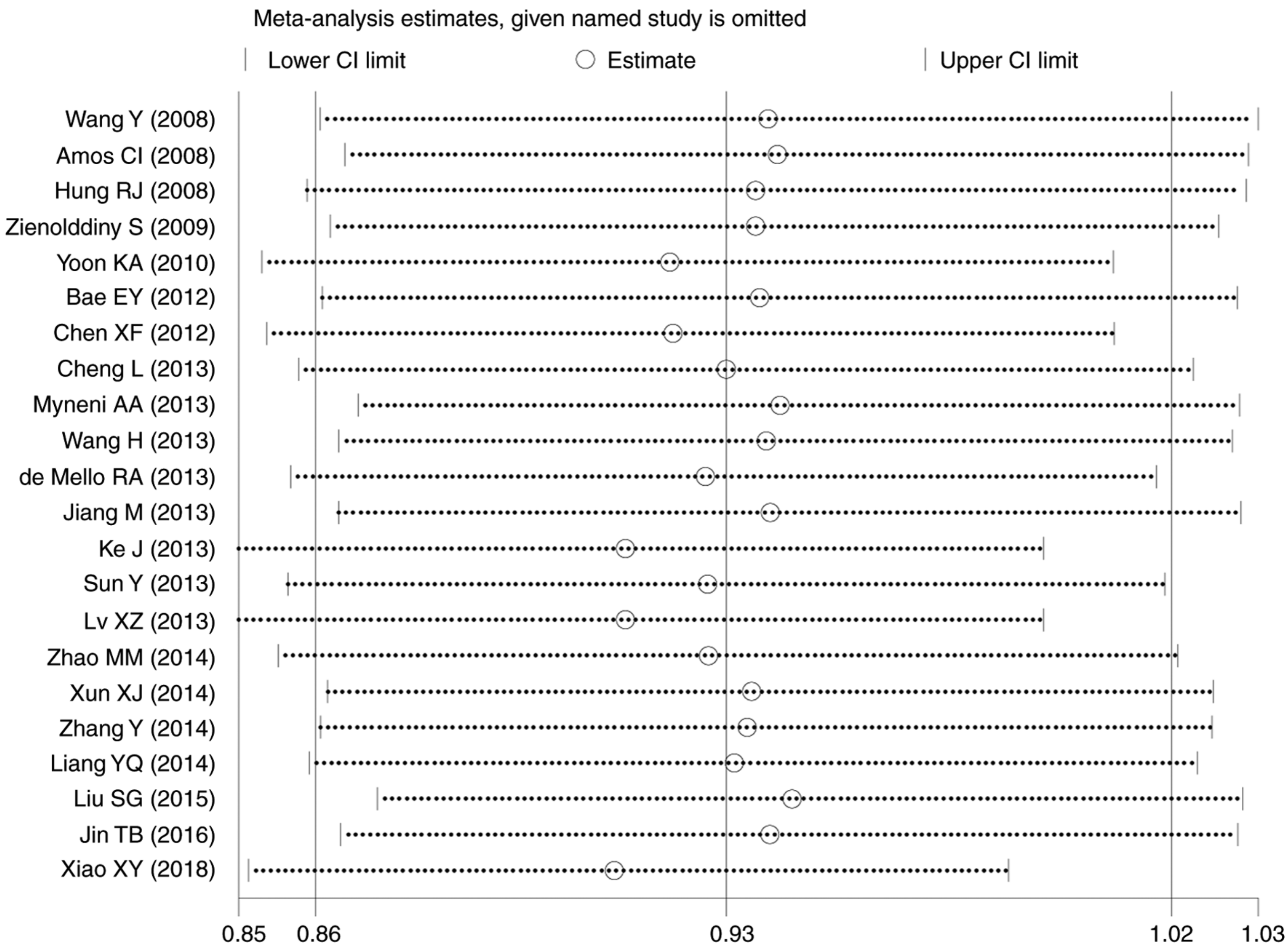

Figure S13. Sensitivity analysis for testing the stability of overall estimates of TT vs. CC + CT.

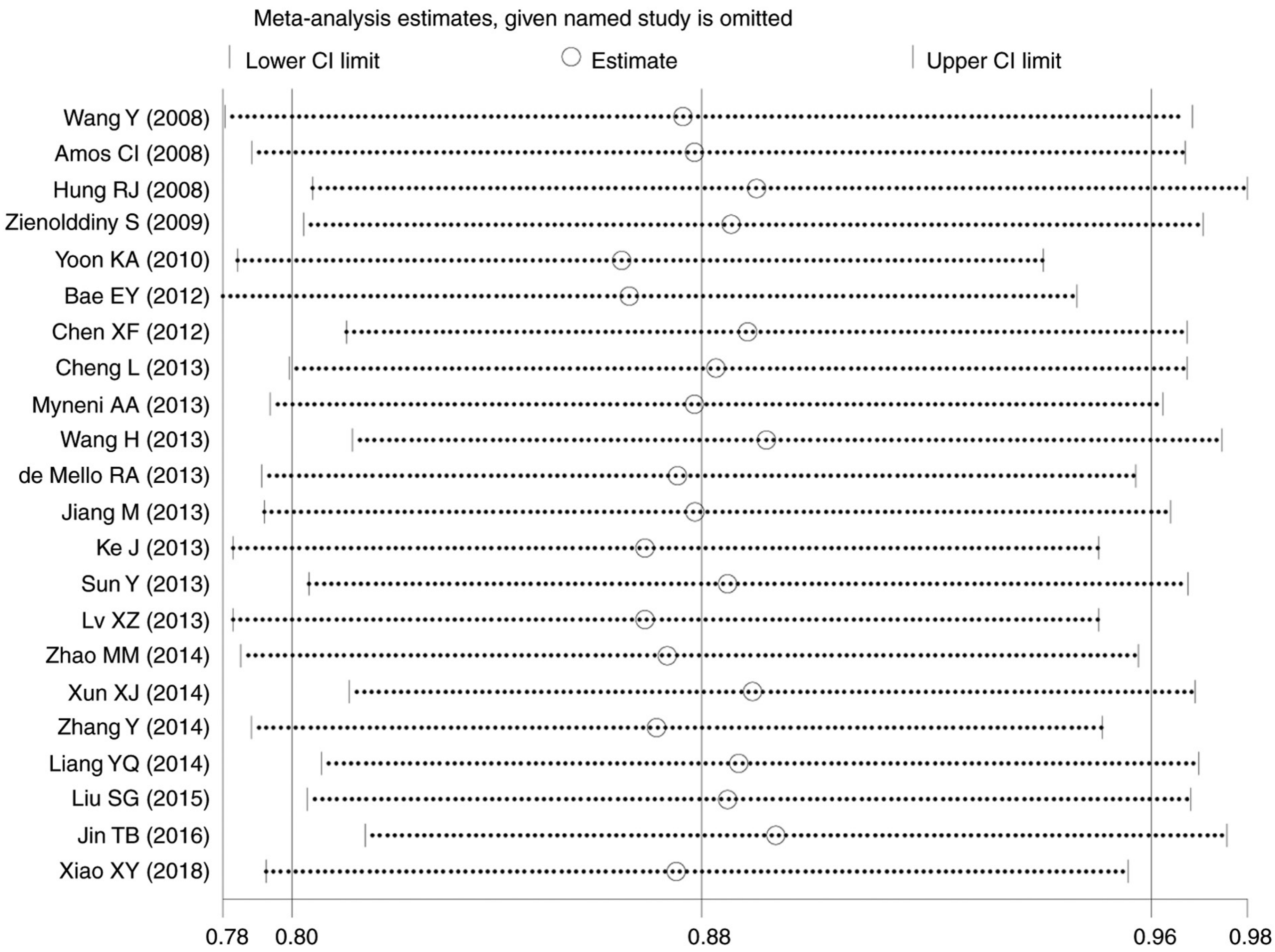

Figure S14. Sensitivity analysis for testing the stability of overall estimates of TT vs. CC.

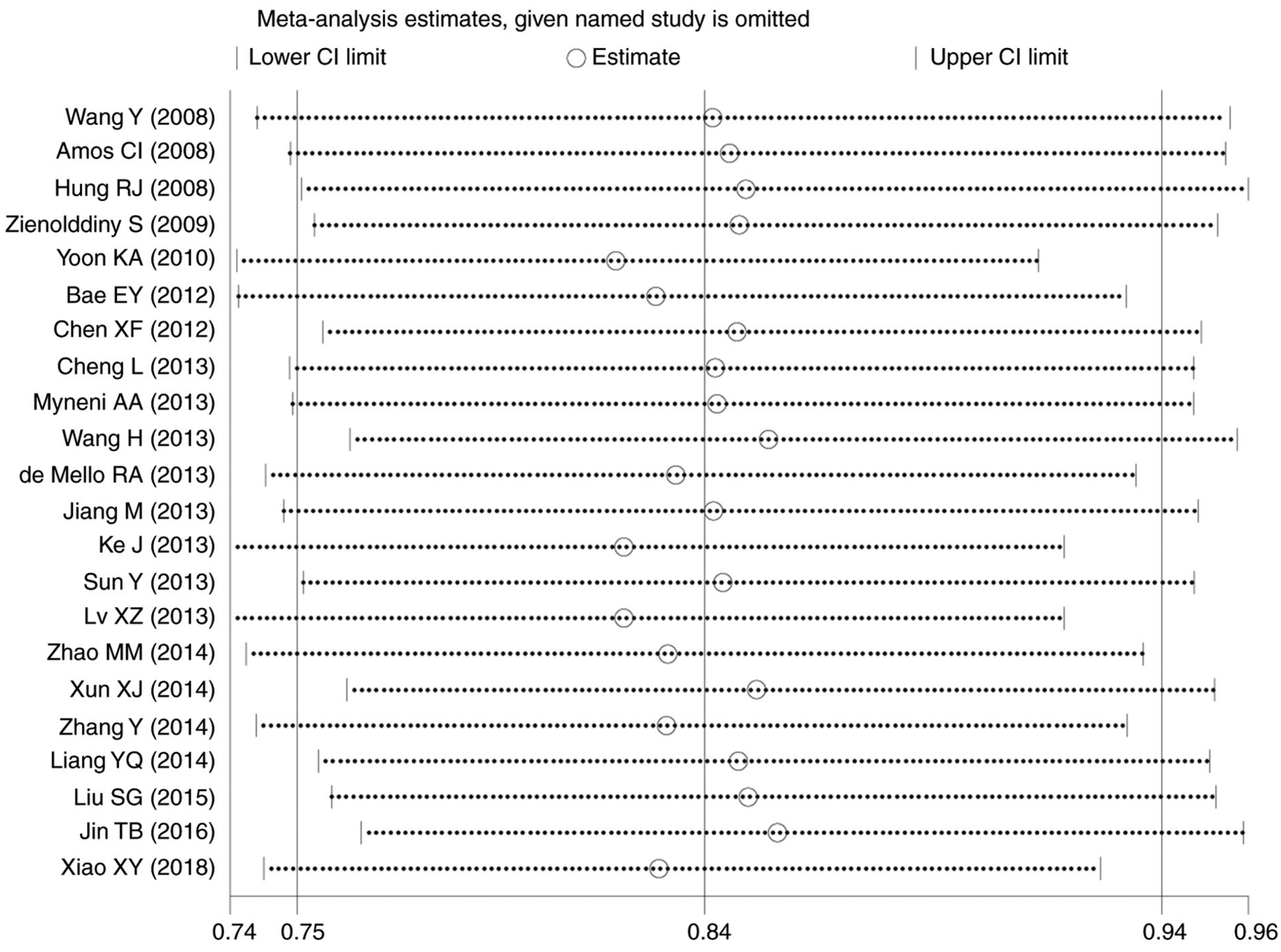

Figure S15. Sensitivity analysis for testing the stability of the overall estimate in the CT vs. CC.

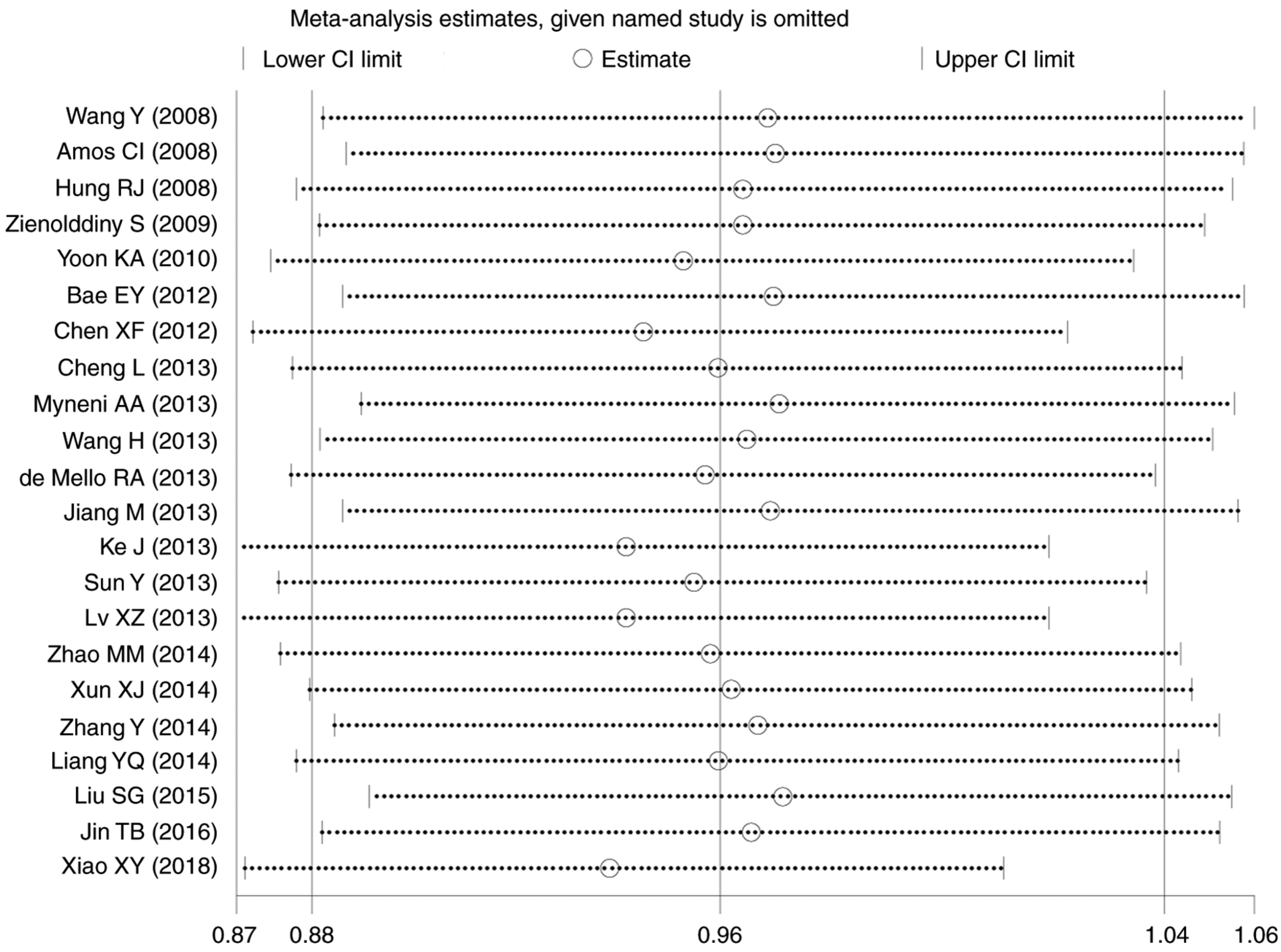

Supplement: Forest plot of the relationship between the cleft-lip and palate transmembrane protein-1-like rs401681 polymorphism and lung susceptibility (including racial subgroup analysis) in the allele T vs. allele C. The squares and horizontal lines correspond to the study-specific OR and 95% CI. The area of  [file Supplementary_Data.pdf]
